# Exercises in Hot and Humid Environment Caused Liver Injury in a Rat Model

**DOI:** 10.1371/journal.pone.0111741

**Published:** 2014-12-30

**Authors:** DongLiang Li, Xiao Wang, Bang Liu, YuZheng Liu, ZhiYu Zeng, LingLing Lu, ZhiYong Zheng, Bing Li, ZongFu Zheng

**Affiliations:** 1 Department of Hepatobiliary Medicine, Fuzhou PLA General Hospital, Fuzhou Fujian, China; 2 Department of Pathology, Fuzhou PLA General Hospital, Fuzhou, Fujian, China; 3 Clinical laboratory, No.476 hospital of PLA Fujian, Fuzhou, Fujian, China; Universidad Pablo de Olavide, Centro Andaluz de Biología del Desarrollo-CSIC, Spain

## Abstract

**Objective:**

To investigate injury pattern during intense exercises in hot and humid environment particularly on liver in a rat exertional heat stroke model.

**Methods:**

We randomly divided 30 rats into a control group (CG), a normal temperature (25±2°C, 60%±5% humidity) exercise group (NTEG) and a high temperature and high humidity (35±2°C, 80%±10% humidity) exercising group (HTEG), each comprising 10 animals. The NTEG and HTEG rats were forced to run in a treadmill for 1 hour maximum at 20 rpm. We analyzed liver cells of all three groups with JC-1 dye and flow cytometry for apoptosis rates in addition to liver tissue 8 - hydroxy deoxyguanosine (8 - OhdG) and blood serum IL–6, tumor necrosis factor alpha (TNF-α), alanine aminotransferase ALT, aspartate amino transferase (AST), serum creatinine (CREA), blood urea nitrogen (BUN), lactate dehydrogenase (LDH), creatine phosphate kinase (CK) concentrations.

**Result:**

Compared with NTEG rats, beside reduced exercise tolerance (60±5 vs. 15±3 minutes) (p = 0.002) the 8-OhdG liver tissue concentrations were significantly higher (p = 0.040) in the HTEG rats. The HTEG developed more organ tissue damage and cellular fragmentations of liver cells. In both exercise groups TNF-α and IL-6 serum concentrations were enhanced significantly (p<0.001) being highest in the HTEG animals. Serum ALT, AST, LDH, CREA, BUN and CK concentrations were significantly enhance in both exercise groups.

**Conclusion:**

In our exertional heat stroke rat model, we found tissue damage particularly in livers during exercises in hot and humid environment that was related to inflammation, oxidative stress and apoptosis.

## Introduction

Heatstroke, which is a clinical emergency with high mortality and morbidity rates, can be divided into classic or non-exertional and exertional heat stroke (EHS). The former, is a clinical syndrome caused by exposure to high temperature and occurs mainly in children, elderly person and those with chronic diseases [1], [2], whereas the latter one, caused by excess heat production over heat loss during intensive exercises, occurs mainly in healthy young people such as athletes and soldiers [3]. Heat strokes are characterized by core body temperatures rising above 40°C and systemic inflammatory responses leading to multiorgan dysfunctions. The incidence of EHS has been increased in recent years and multiple organ failure may occur in serious cases, which are life threatening [4]. At present, general safety parameters in hot and humid environments are not established and the pathogenesis of multiple organ damage resulting from EHS is not entirely clear. In the early 1900 s, studies noted, that the clinical manifestation of heat stroke was similar to sepsis [5]. Lipopolysaccharides (LPS) serum concentrations rose significantly up to 8.6 ng/ml in heat stroke patients, which was significantly higher than the conventional lethal concentration of 1 ng/ml [6]. This has also been confirmed in animal EHS experiments, in which the plasma concentration of lipopolysaccharides in an experimental group was 4.8 times (2.9–7 times) higher than in the control group and sepsis treatment could improve heat tolerance and the survival rates of heat exposed animals. After pretreatment with LPS antibodies, all monkeys survived body temperatures of 43.5°C, whereas 5 of 6 control monkeys died. In the dead animals of the control group, plasma LPS levels were significantly enhanced compared to the LPS antibody treated primates and the one control monkey, which survived [7]. Heat stress induces cutaneous vasodilatation and splanchnic vasoconstriction, leading to splanchnic hypoperfusion and ischemia, resulting in increased production of reactive oxygen and nitrogen species, which may in turn induce intestinal mucosal injury and hyperpermeability for then circulating endotoxins like LPS [8]. LPS are mainly decomposed by neutrophils, eosinophils and basophils as well as liver Kupffer cells, which release then IL-1, IL-6, TNF-α and other inflammatory cytokines that attract leukocytes and trigger further inflammatory processes eventually leading to a systemic inflammatory response syndrome and local tissue injuries particularly of internal organs [9]–[11]. Hsie et al. (2011) detected after 43°C heat stress for 68 minutes in rats hypothalamic neuronal degeneration and apoptosis with serum concentration increases of TNF-α, intercellular adhesion molecule-1 (ICAM-1) and interleukin-10 (IL-10) accompanied by spleen, liver, kidney, and lung cell apoptosis [12]. However, heat stroke may also be caused by direct heat effects on tissue cells, like cell membrane damages, especially at elevated temperatures [13]. Intense exercises can induce liver tissue ischemia, reperfusion and oxidative stress with free radical activity and we hypothesized that differences between exertional and non-exertional heat strokes might be detectable in liver and other organ damage caused by exercise. In the present study, we compared effects of exercises alone and in combination with heat on physiological and morphological changes in a rat model.

## Materials and Methods

### Experimental animals

40 Sprague - Dawley rats, 7-week-old, weighting 150–200 gram were provided by the Shanghai Xitang Biological Technology center and raised in the animal experiment center of the Fuzhou General Hospital of the Nanjing military region under room temperature of 25±2°C and humidity of 60%±5%, lighting for 12 hours per day with circadian rhythm changes. They were fed by sterilized fodder and water ad libitum. The study was approved by the ethical committee of the Fuzhou General Hospital and the care of mice complied with the Beijing Administration Rules of Laboratory Animal handling (GB 14925-2001).

### Experimental design

All rats were trained for load-increasing runs for 5 days. The length of the treadmill (Type: XR-YLS-15A, Shanghai Xinqing soft information technology co., LTD, Shanghai, China) belt was 650 cm. The initial speed of the running machine was 10 RPM ( = 0.39 km/h) with a training time of 10 minutes, which was increased to 15 RPM ( = 0.585 km/h) and 15 minutes in the next day, followed by 2 RPM ( = 0.078 km/h) and 5 minute increases each day to finally 21 RPM ( = 0.819 km/h) and 30 minutes running time on the 5^th^ day. After the 5 day exercise period, rats which could not adapt to the running machine or had injuries of sole or foot were excluded and 30 rats eligible for exercises after training were randomly divided into 3 groups: 10 rats were the CG, 10 rats comprised the NTEG and 10 rats were selected for the HETG. All rats rested for 24 hours after 5 days of training and the experiment was performed on the 7^th^ day. Rats in the CG were kept in the animal rooms under normal temperature (25±2°C) and humidity (60%±5%). Rats in the NTEG and HETG were tested at normal conditions (25±2°C, 60%±5% humidity) and in high temperature and high humidity (35±2°C, 80%±10% humidity), respectively. The speed of the treadmill was adjusted to 20 RPM ( = 0.78 km/h) and electric shocks with a limit of 1.00 mA were applied with intervals of 30 seconds after one shock for keeping the animals running. The body temperature was anally measured every 10 minutes in the NTEG and every 3 minutes in the HTEG animals. EHS was diagnosed at the time when deep breathing with cyanosis of the limb occurred and 3 or more electric shocks were necessary within 60 minutes of the experiment with body temperatures reaching between 42.5°C and 43°C. The experiment lasted from the exercise initiation time to the occurrence of heat strokes, which was recorded automatically by a supporting treadmill software.

### Specimen collection

After running, rats that met the criteria of EHS as well as the control group were anesthetized with intraperitoneal injections of 0.5 ml/kg phenobarbital sodium and blood samples were collected. After cervical dislocation under anesthesia, the internal organs heart, lung, liver, kidney as well as the brains were gathered and rinsed clean in 0.9% saline for further analyses.

### Hepatocyte mitochondrial transmembrane potential detection

Fresh liver tissues of about 100 mg were cut into pieces of 1 mm^3^ and PBS was added. After blending, the suspensions were filtered with 200 mesh sieves and the filtrate contained liver cells. The cells were collected at a concentration of 1×10^6^ cells in 500 µL 1 × incubation buffer to which 10 µl of JC −1 working fluid was added. Then the liver cells were incubated for 15–20 min in a 5% CO_2_ incubator at 37°C, collected by centrifugation at room temperature (2000 RPM, 5 min) and after 1 time washing suspended in PBS. We used a flow cytometry instrument (BD FACSCalibur, San Jose, CA, US) (Ex = 488 nm; Em = 530 nm) to detect5, 5′, 6, 6′-tetrachloro1, 1′, 3, 3′-tetraethyl-benzimidazolylcarbocyanine iodide (JC-1; Molecular Probes, Eugene, OR) staining, which indicates the state of mitochondrial transmembrane potential. Polarized mitochondria appear as red and depolarized mitochondria as green. Green fluorescence was detected through the FITC channel FL1; Red fluorescence was detected through the PI channel FL2. Apoptosis was indicated by an increase in the green/red fluorescence intensity ratio [14].

### Detection of 8 - hydroxy deoxyguanosine (8 - OhdG) in liver tissues

100 mg liver tissues were quickly homogenized on ice with a glass homogenizer, dissolved in 1 ml PBS and then centrifuged for 20 minutes (2000 RPM). The resulting supernatant was further processed according to the manufacturer’s protocol (8-OhdG ELISA assay, Xikang, Shanghai). The final values were measured at 490 nm in a microplate reader and calculated based on an 8-OhdG concentration standard curve.

### Plasma IL – 6 and TNF α level detection

The blood serum IL – 6 and TNF-α concentrations were determined with (IL–6 ELISA, TNF- α ELISA kits) (Qiaoyi, Shanghai, China) according to the manufacturers’ protocols and are shown as pg/ml. Other blood serum analyses included ALT (related to liver), aspartate amino transferase (AST) serum creatinine (CREA), blood urea nitrogen (BUN), lactate dehydrogenase (LDH), creatine phosphate kinase (CK) and were detected with common hospital laboratory equipment.

### The histopathological analyses of liver, kidney, heart, lung, and brain tissues

First, the tissues were fixed in 10% formalin for 72 hours and then cut into appropriate sized pieces for dehydration. After that, the tissues were incubated in transparent xylene agent and then immerged into a mixture of transparent agent and paraffin wax until the paraffin fully replaced xylene. The wax blocks were then fixed in a microtome, sliced into 5–8 mm thick sections and immerged into hematoxylin and 1% eosin staining solution. After de-waxing, we observed histopathological changes under a light microscope.

### Liver tissue electron microscopic sample preparation and observations

The liver tissues were cut into 1 mm^3^ sized blocks and fixed for at least 2 hours in 3% glutaraldehyde plus 1.5% paraformaldehyde in 0.1 M PBS (pH7.2) at 4°C. Then we rinsed and fixed them with 1% osmic acid −1.5% potassium ferrocyanide and after dehydration cut them into 100 nm ultrathin slices with a Leica UC-6 ultra-microtome. Finally, the samples were stained with uranium and lead citrate for 5 to 15 minutes, washed with distilled water and observed with a transmission electron microscope (PHILIPS EM208, FEI Company, Eindhoven, Netherlands).

### Statistical analyses

All data were analyzed with the SPSS13.0 statistical software package. Group measurement data are presented as mean ± standard deviation (±sd) and comparison among groups was performed using one-way analysis of variance (One - way ANOVA). P values <0.05 were considered statistically significant.

## Results

### The running time in a hot and humid environment was less than in normal temperature

The average running time in the NTEG was 60±5 min and 15±3 min in the HTEG, with significant differences between them (F = 7.232, P = 0.002).

### The mitochondria depolarized in hepatocytes after exercise under the condition of high temperature and high humidity

**Figure 1:**
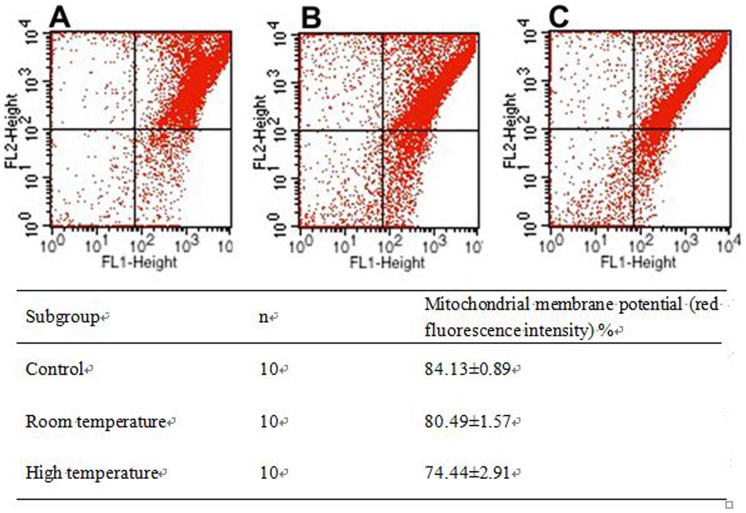
Flow cytometric histogram and percentage of rat liver cell apoptosis rates detected by JC-1 dye. A) Control, B) NTEG, C) HTEG (room temperature vs. control group: p = 0.006; high temperature vs. control group: p = 0.002; room temperature vs. high temperature: p = 0.040); FL1 = green fluorescence, FL2 = red fluorescence.

As shown in Fig. 1, the flow cytometric analyses revealed a significant increase of mitochondrial depolarization in cells derived from heat stress rats.

### Biochemical parameter changes

Serum concentration of ALT, AST and LDH, CREA, BUN and CK were significantly different among all groups. Compared with the HTEG, ALT (P<0.05), LDH (P<0.05) and CK (P<0.05) serum concentrations were significantly higher, whereas AST (P<0.05) was lower in the NTEG. BUN and CREA (related to renal functions) analyses revealed that the former was higher concentrated in sera of the NTEG (P<0.05) and the latter was higher concentrated in sera of the HTEG (P<0.05) (Table 1).

**Table 1:** Comparison of serum changes in the rat groups.

	Control	Room temperature (NTEG)	High temperature (HTEG)
Liver function and lactate dehydrogenase			
ALT(U/L)	52.8±4.8	76.9±15.2*	71.3±16.7**Δ
AST(U/L)	162.0±36.0	216.0±61.7**	279.2±79.12***Δ
LDH(U/L)	1215.5±237.0	1835±451.6	1466.8±445.5Δ
Kidney function and creatine kinase			
CREA (µmol/L)	26.1±3.9	48.2±16.9**	54.2±17.0***Δ
BUN (mmol/L)	8.2±1.3	16.5±4.9***	12.0±1.2***Δ
CK(U/L)	1981.4±577.9	3580.7±543.2**	3319.9±1431.5*Δ

Compared to control group,*p<0.05, **p<0.01, ***p<0.001. Comparisons of NTEG and HTEG, Δp<0.05, ΔΔ<0.01, ΔΔΔp<0.001.

### Changes of 8-OhdG in rat liver tissues and inflammatory related cytokines in blood plasmas

The liver 8-OhdG (P<0.05), IL-6(P<0.05) and TNF- α (P<0.05) plasma concentrations were statistically significant and gradually higher in NTEG and HTEG rats compared to the control (Table 2).

**Table 2:** Comparison of the changes between groups of 8OhdG in the liver tissue, and serum inflammation factors (IL-6, TNF-a) in the rat.

	Number	8OhdG (pg/ml)	IL-6 (pg/ml)	TNF-α (pg/ml)
Control	10	1923.5±242.3	255.0±138.8	44.8±48.4
Normal Temperature (NTEG)	10	2038.3±418.2*	427.4±121.3*	226.3±71.5***
High temperature (HTEG)	10	2401.3±476.3*Δ	534.2±152.3***Δ	253.9±94.7***Δ

Compare to control group,*p<0.05, **p<0.01, ***p<0.001; Comparison of NTEG and HTEG, Δp<0.05, ΔΔp<0.01, ΔΔΔp<0.001.

### Exertional heat stress led to morphological organ tissue alterations

**Figure 2:**
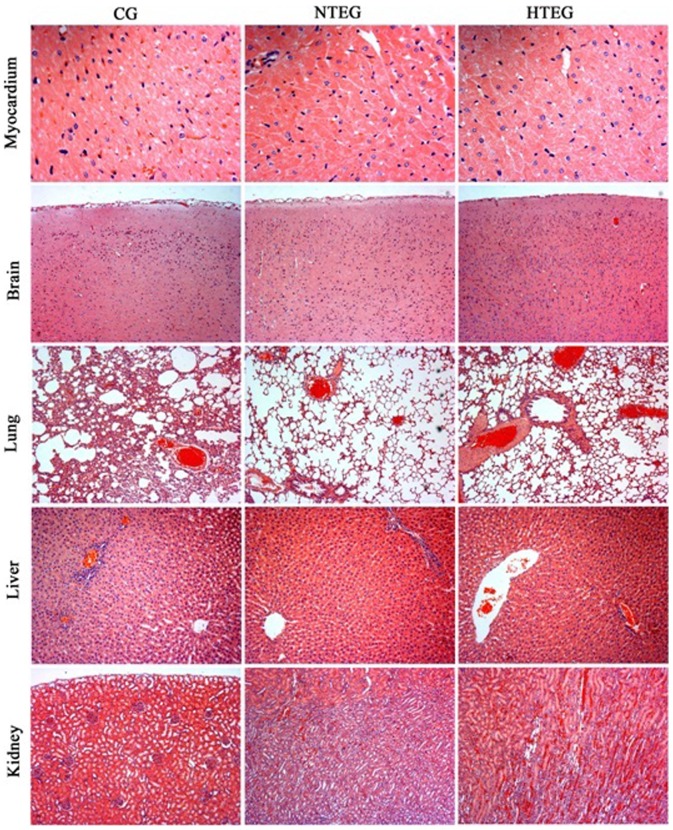
Histopathological changes in major organs. Microscopical images of H&E stained rat myocardium, brain, lung, liver and kidney tissue sections from control group (CG), normal temperature exercise group NTEG (middle panel) and high temperature exercise group (HTEG) (right panel) (magnification x50).

Next, we analyzed whether organ tissue structures were affected by the exercises. As visible in Fig. 2, various histological changes in different degrees occurred during the exercises. **Myocardium**: Compare to control group, in the NTEG we found a mild swelling of myocardial cells, while in the HTEG the swelling of myocardial cells was increased obviously. **Brain:** In the NTEG, the grey matter in the cerebellum contained no obvious lesions as well as no congestion in the blood vessels of the cortex, but in the HTEG, endovascular blood clots appeared mainly in the cortex. **Lungs**: Compared to control group, in both exercise groups the pulmonary alveoli were dilated with hyperinflation. In addition, alveoli walls became thin and ruptured forming pulmonary bullae and arterial blood clots appeared. **Liver**: In the NTEG we found mild cell edemas and increased cell volume, while in the HTEG, we detected more obvious liver cell edemas with expansion of central veins and hepatic vein blood clots. **Kidney**: Compared to the control group, in the NTEG, we found no obvious changes in renal glomeruli, but mild blood clots in the renal tubulointerstitial area and medulla. In the HTEG, the epithelial cells of the renal proximal convoluted tubules appeared cloudy swollen and stromal as well as in medullar intravenous congestions were visible.

### Liver cells lost their structures upon exercises in hot and humid environment

**Figure 3:**
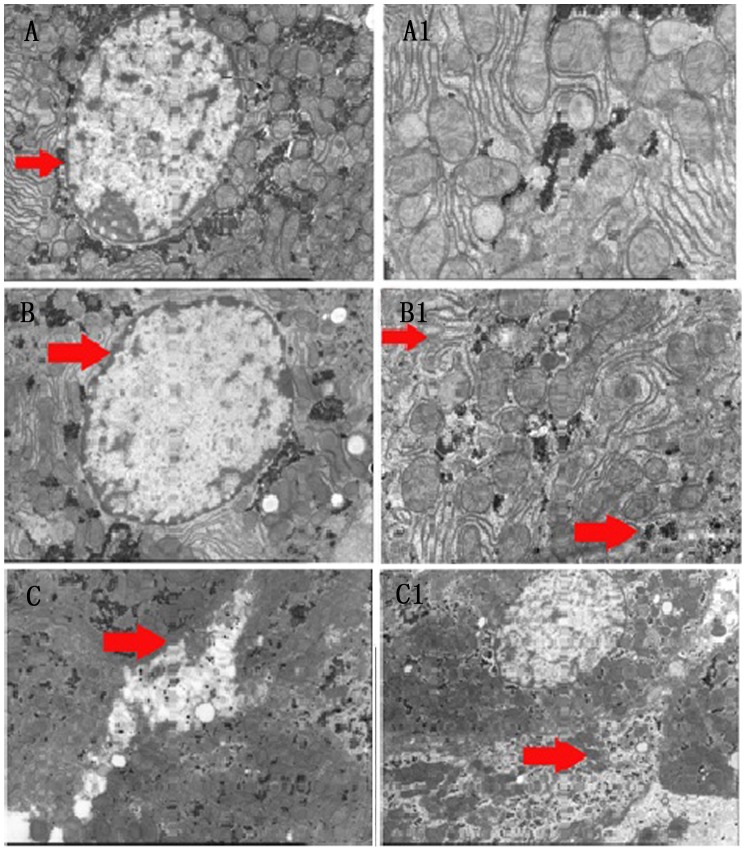
Electron microscope images of rat liver cell changes. Control group: (A) large and round nucleus in the center with smooth nuclear membrane (arrow), visible nucleolus and rich in shallow euchromatin. (A1) The cells had a neat structure with clear and complete mitochondrial organization. NTEG: (B) Nucleus with thickened and dissolved boundaries of the nuclear membrane (arrow). (B1) The cellular structures became unclear and mitochondrial cristae changed their shapes (upper arrow). The endoplasmic reticulum and other organelles became diffuse (lower arrow). HTEG: (C) membrane rupture, scattered organelles outside the cell (arrow), C1) disappearance of normal cell organization and dissolved mitochondrial structures (arrow).

In order to investigate particularly liver tissue damages, we analyzed liver cell structures with electron microscopy. Cell organelles dissolved and mitochondrial structures as well as normal cell organization disappeared and became incoherent, particular in the HTEG (Fig. 3).

## Discussion

In our rat model study, we induced heat strokes by forced extreme exercises in a hot and humid environment and observed changes of blood biochemistry, organ histology, inflammatory responses as well as enhancements of the oxygen radical indicator 8-OhdG and changed liver cell mitochondrial structures. 8-OhdG is an oxidized derivative of deoxyguanosine and the major product of DNA oxidation under oxidative stress [15]. Our 8-OhdG results are in line with previous literature about oxidative stress in human livers after swimming [16] and in over exercised rats [17], which further confirmed that oxidative stress is occurring during exertional heat stroke. Because oxidative damage occurs 3 times more in mitochondria than in the cytosol since the mitochondria are the major source of free oxygen radicals [18] the enhanced 8-OhdG concentrations also reflected in mitochondrial depolarization, indicating liver cell apoptosis in the HTEG (Fig. 1). In our study, liver was the major affected organ of heat stress damage, probably because of its central body location and accelerated metabolic rate at temperature rises [19], [20]. It is known that after physical exercises ALT, AST, LDH and CK serum levels are significantly enhanced [16], [21], [22], which we also detected in both exercise rat groups (Table 1). AST, which serum concentration was higher in the HTEG, is mainly located in the mitochondria and the de Ritis ratio (AST/ALT) as measure for liver damage severity [23] was higher in the HTEG (3.9 vs. 2.8) than in the NTEG, both indicating that liver and particularly mitochondrial damage was more obvious in the HTEG. Lower LDH and CK serum concentrations in the HTEG might reflect the reduced average running time in the HTEG compared to the NTEG (15±3 vs. 60±5 min). BUN was higher concentrated in the NTEG, whereas CREA was higher concentrated in sera of the HTEG (P<0.05). We suggest, that in the rats which exercised for an extended time in normal temperature, increasing protein decomposition occurred and more pronounced elevated BUN serum concentrations developed [24], while in the heat exercise rats, multiple organ failure included acute renal failure causing in turn obviously strong elevated CREA concentrations, which were reported to be mortality indicators for heat stroke patients [25].

Proinflammatory cytokines such as TNF- α and IL-6 in high temperature exercising rats increased significantly compared to the normal temperature exercise and control groups, which is in accordance with previous literature of 30-fold serum IL-6 concentration increases after prolonged ultra-endurance exercises [26]. Exercise related splanchnic hypoperfusion as a cause of intestine damages [27] is similar to heat stroke induced intestinal mucosal injury and hyper-permeability changes, which leads to leakage of endotoxins and increased production of inflammatory cytokines such as TNF- α like during sepsis [8]. A high plasma TNF-α level can be considered as a possible trigger of systemic inflammatory responses via stimulating the release of other cytokines such as IL –4 and IL –6, which eventually may expand the initial biological effect to systemic progression of the inflammatory response, thus leading to the occurrence of a multiple organ dysfunction syndrome [28]–[30].

Taken together, our results suggested that exercising in hot and humid environment could further aggravate particularly liver damage and induce hepatic failure. During strenuous exercises liver blood flow decreased leading to liver ischemia/hypoxia and increases of free nitrogen and oxygen radicals, lipid peroxidation and inflammation with high cytokine IL-6 and TNF-α releases caused by activated intrahepatic Kupffer cells finally resulting in complete liver cell damage.

Conclusion: The results of our study revealed, that levels of TNF- α and IL-6 increased significantly in rats exercising under heat and high humidity, suggesting that this conditions induced a systemic inflammatory response syndrome which caused multiple organ damages. At the same time, the mitochondrial membrane potential in liver cells decreased whereas the content of 8-hydroxy deoxyguanosine acid increased in the heat stress exercise rats, suggesting that lipid peroxidation and oxidative stress induced by tissue ischemia/hypoxia played an important role in liver injury when extended exercises and heat were combined in our EHS rat model.
